# Hydrothermal synthesis of CuO@MnO_2_ on nitrogen-doped multiwalled carbon nanotube composite electrodes for supercapacitor applications

**DOI:** 10.1038/s41598-022-16863-3

**Published:** 2022-09-20

**Authors:** Vijay Kakani, Sivalingam Ramesh, H. M. Yadav, Chinna Bathula, Praveen Kumar Basivi, Ramasubba Reddy Palem, Heung Soo Kim, Visweswara Rao Pasupuletti, Handol Lee, Hakil Kim

**Affiliations:** 1 Department of Integrated System Engineering, Inha University, 100 Inha-ro, Nam-gu, 22212 Incheon, Republic of Korea; 2 Department of Mechanical, Robotics and Energy Engineering, Dongguk University-Seoul, Pil-dong, Jung-gu, 04620 Seoul, Republic of Korea; 3 School of Nanoscience and Bio-Technology, Shivaji University, Kolhapur, 416004 India; 4 Division of Electronics and Electrical Engineering, Dongguk University-Seoul, 04620 Seoul, Republic of Korea; 5 Department of Chemistry, Sri Venkateswara University, Tirupathi, Andhra Pradesh 517502 India; 6 Department of Medical Biotechnology, Dongguk University, 10326 Gyeonggi, Republic of Korea; 7 Department of Biomedical Sciences & Therapeutics, University Malaysia Sabah, 88400 Kota Kinabalu Sabah, Malaysia; 8 Department of Biochemistry, Abdurrab University, Jl Riau Ujung No. 73, Pekanbaru, 28292 Riau, Indonesia; 9 Department of Environmental Engineering, Inha University, 100 Inha-ro, Nam-gu, 22212 Incheon, Republic of Korea; 10 Department of Electrical and Computer Engineering, Inha University, 100 Inha-ro, Nam-gu, 22212 Incheon, Republic of Korea

**Keywords:** Chemical synthesis, Supercapacitors

## Abstract

Nitrogen-doped multiwalled carbon nanotubes (N-MWCNTs) have been used to fabricate nanostructured materials for various energy devices, such as supercapacitors, sensors, batteries, and electrocatalysts. Nitrogen-doped carbon-based electrodes have been widely used to improve supercapacitor applications via various chemical approaches. Based on previous studies, CuO@MnO_2_ and CuO@MnO_2_/N-MWCNT composites were synthesized using a sonication-supported hydrothermal reaction process to evaluate their supercapacitor properties. The structural and morphological properties of the synthesized composite materials were characterized via Raman spectroscopy, XRD, SEM, and SEM–EDX, and the morphological properties of the composite materials were confirmed by the nanostructured composite at the nanometer scale. The CuO@MnO_2_ and CuO@MnO_2_/N-MWCNT composite electrodes were fabricated in a three-electrode configuration, and electrochemical analysis was performed via CV, GCD, and EIS. The composite electrodes exhibited the specific capacitance of ~ 184 F g^−1^ at 0.5 A g^−1^ in the presence of a 5 M KOH electrolyte for the three-electrode supercapacitor application. Furthermore, it exhibited significantly improved specific capacitances and excellent cycling stability up to 5000 GCD cycles, with a 98.5% capacity retention.

## Introduction

Recently, electronic devices for storage applications comprising nanometer-scale materials with excellent capacitance and cyclic stability are being rapidly developed. Numerous methods for fabricating electrode materials for practical applications have been reported in various supercapacitor, battery, and fuel-cell studies^1–4^. In particular, supercapacitors have received considerable attention owing to their excellent life cycles and high-power density results. Supercapacitors can be categorized as electric double-layer capacitors (EDLC) and pseudocapacitors, depending on their complex electrochemical reaction. In the case of the EDLCs, energy is collected by the electrostatic adsorption of charges on the electrode surface in the parallel plate capacitor. In pseudocapacitors, energy is collected via reversible Faradaic responses on the material electrode surface^5–8^.

Carbon-based electrode materials are an appropriate choice for supercapacitor applications because of their excellent specific capacitance and power density^9–11^. An alternative approach is to use potential electrode materials such as Co_3_O_4_, MnO_2_, NiO, CuO, Fe_3_O_4_ and V_2_O_5_, which have been widely used in supercapacitor applications^12–15^. The higher specific capacitance of these materials is because of the presence of metal oxides, which exhibit more pronounced redox behavior than the carbon in carbon-based electrodes; thus, a specific capacitance and excellent electrochemical stability are realized via irreversible reactions^16–18^. Notably, copper is an excellent electrode material because it is nontoxic, abundantly available, low-cost, and easy to fabricate into an electrode for supercapacitor applications^17–19^. Zhang et al. reported that flower-like CuO nanostructured materials yielded a capacitance value of 134 F g^−1^ in the presence of a 1 M KOH electrolyte^18^. In addition, mesoporous copper oxide nanoribbons were fabricated on nickel foam electrodes, with a capacitance of 137 F g^−1^. Wang et al. fabricated nanosheets with a capacitance of ~ 569 F g^−1^^19^.

MnO_2_ has emerged as a promising electrode material for supercapacitor applications owing to its high theoretical specific capacitance, high electrochemical activity, and environmental friendliness^20–22^_._ However, the low conductivity (10^−5^ to 10^−6^ S cm^−1^) and unstable structure of MnO_2,_ which causes poor electrochemical cyclability, limits its application. To address these limitations and improve the electrochemical behavior of supercapacitors, several studies have attempted to design nano-MnO_2_ structures. It has been reported that the electrochemical performance of MnO_2_ depends on its morphology, surface area, and crystal structure. Zhang et al. reported MnO_2_-based electrodes for supercapacitors with different crystal structures and morphological behaviors synthesized via a hydrothermal process. Nano-a-MnO_2_ ball with a low degree of crystallinity exhibited a high specific capacitance of 200 F g^−1^ and excellent cyclic stability^.^ Rusi et al. also synthesized a-MnO_2_ with a low-crystalline electrode via electrochemical deposition using a manganese acetate tetrahydrate electrolyte. The assembled MnO_2_ electrode, characterized via a cyclic voltammetry test in the Na_2_SO_4_ electrolyte at a scan rate of 1 mV s^−1^, exhibited a high specific capacitance of 238 F g^−1^ and excellent stability, with an 84% capacitance retention over 1900 cycles. By contrast, CuO, which is an active material that contributes to pseudocapacitance, possesses a high specific surface area, is highly conductive and environmentally friendly, and can be incorporated with MnO_2_. Furthermore, the synergetic effect also plays an important role in improving the performance of the MnO_2_–CuO composite on the carbon surface. Therefore, a material comprising both MnO_2_ and CuO will exhibit better electrochemical performance, conductivity, cycling stability, and morphological properties than individual MnO_2_ and CuO materials. Therefore, this study focused on CuO@MnO_2_ and CuO@MnO_2_/N-multiwalled carbon nanotube (MWCNT) composites synthesized via a hydrothermal reaction process using a three-electrode configuration for supercapacitor applications. The nanocrystalline structure and morphological properties of the resulting composites were investigated via FE-SEM. In addition, the electrochemical properties of the composite electrodes were analyzed using CV, GCD, and EIS in the presence of a 5M KOH electrolyte.

## Experimental methods

### Materials

MWCNTs, copper nitrate penta hydrate Cu(NO_3_)_2_·5H_2_O, potassium permanganate (KMnO_4_), manganese (II) acetate (C_4_H_6_MnO_4_), hydrochloric acid (HCl), sulfuric acid (H_2_SO_4_), phosphoric acid (H_3_PO_4_), ammonia (NH_3_, 30%), absolute ethanol (C_2_H_5_OH), *N*-methyl pyrrolidinone (NMP), and polyvinylidene fluoride (PVDF) were obtained from Sigma-Aldrich Chemicals, and the overall electrochemical experiments were performed using double distilled (DD) water.

### Synthesis of CuO@MnO_2_/N-MWCNT composite

**Figure 1:**
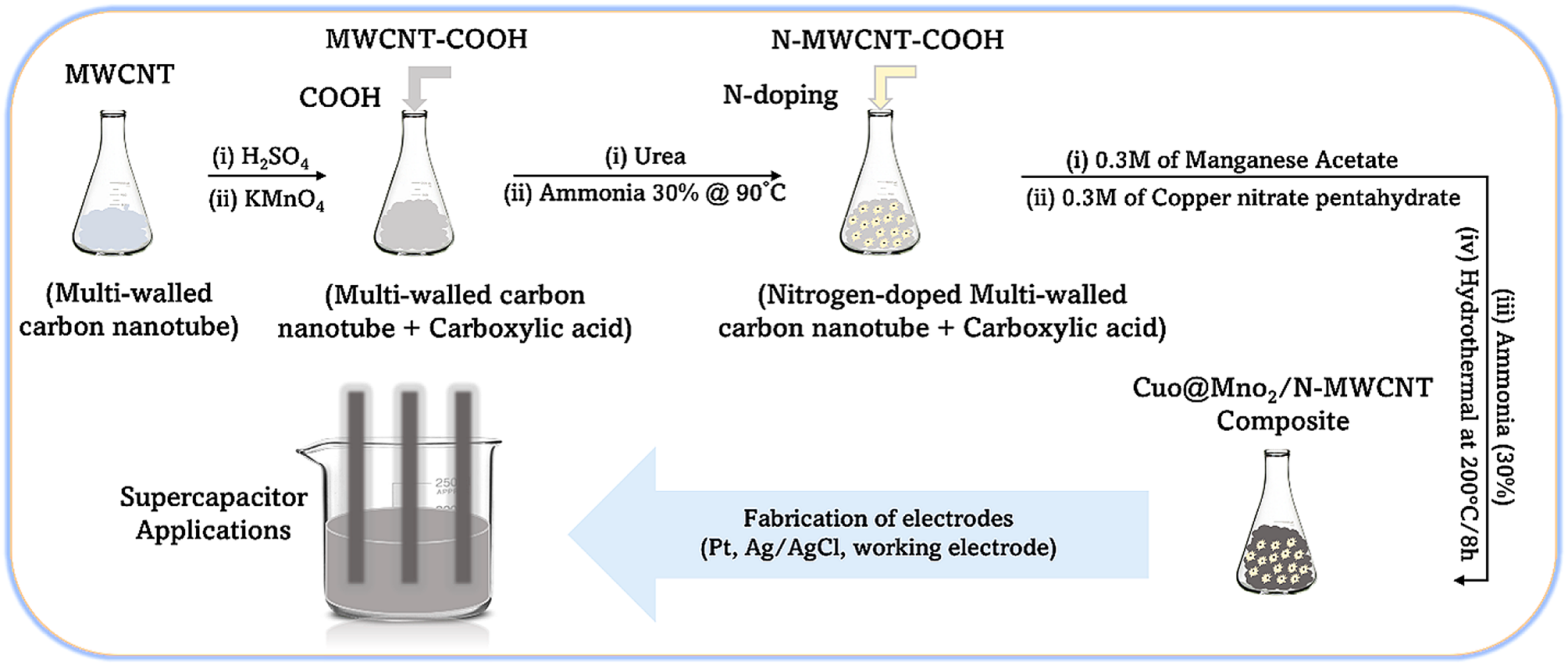
Schematic of the CuO@MnO_2_/N-MWCNT composite synthesis process.

The required MWCNT and nitrogen-doped MWCNT syntheses are described in our previous reports^20,21^. In summary, to synthesize the CuO@MnO_2_ composite, 0.65 of N-MWCNTs was diffused in 200 mL of double DD water via sonication for 2 h to achieve complete dispersion of the tubes. For this, 0.3 moles of copper nitrate CuSO_4_·5H_2_O and 0.3M manganese acetate/KMnO_4_ were added, followed by 20 mL of 30% ammonia, and the solution was stirred at 90 °C for 12 h. At this point, the reaction mixture was transferred to an autoclave reactor, and the hydrothermal reaction was carried out at 200 °C for 8 h. The precipitated CuO@MnO_2_/N-MWCNT composite material was filtered and washed repetitively with a 1:1 solution of DD water/ethanol and purified at 90 °C for 12 h. The dried composite was stored in an airtight bottle and subjected to structural, morphological, and electrochemical analyses. A schematic of the CuO@MnO_2_/N-MWCNT composite synthesis is shown in Fig. 1.

### Fabrication of electrodes for supercapacitor study

The composite was fabricated via a three-electrode configuration and its electrochemical properties were determined using CV, GCD, and EIS analyses. The material (85:15:5) was mixed with *N*-methyl pyrrolidinone (NMP) using a uniform paste. Subsequently, this composite paste was coated uniformly on a strip of a nickel wire (1 × 1 cm^−2^) current collector and vacuum-dried in an oven at 90 °C for 10 h. The fabricated working electrode, Pt electrode (counter electrode), and a reference electrode (Ag/AgCl) were used to study the electrochemical properties of the synthesized composite materials.

### Materials characterization

The synthesized CuO@MnO_2_ and CuO@MnO_2_/NMWCNT composite materials were characterized using Raman, XRD, SEM, SEM–EDS, TEM, and CV analyses for supercapacitor application. XRD results of the composite materials were obtained using a Rigaku Rotaflex (RU-200B) X-ray diffractometer. The composite materials were analyzed using a He–Ne laser beam in the RM 200 Raman spectral microscope, and the morphological properties of the composite samples were determined using FE-SEM (JEOL) and SEM–EDX analyses. The electrochemical properties of the composite materials were determined based on CV, GCD and EIS results via CHI 7081C (CH Instruments, workstation Inc., USA).

## Results and discussion

### Structural properties

**Figure 2:**
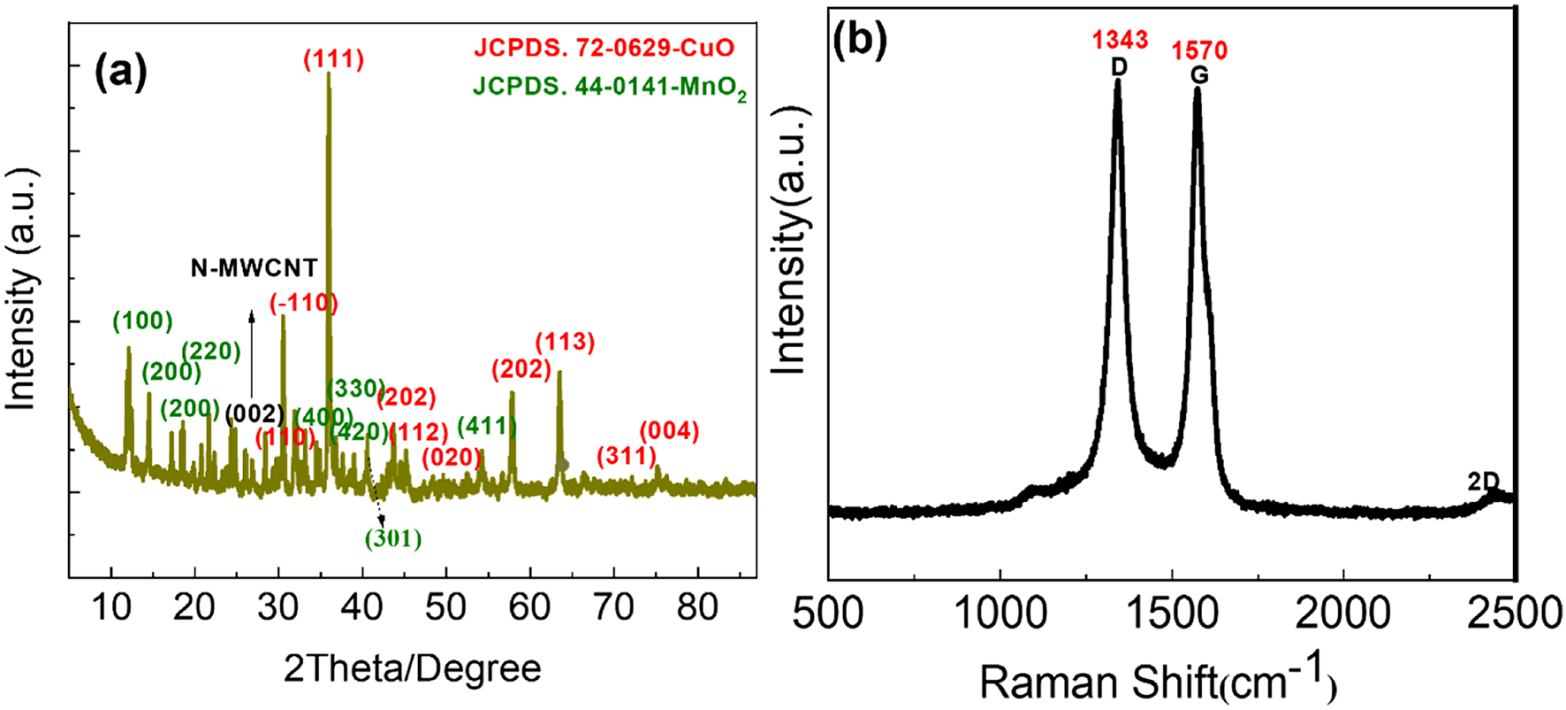
(**a**) XRD and (**b**) Raman spectra of the CuO@MnO_2_/N-MWCNT composite.

Figure 2a shows the diffraction patterns of the CuO@MnO_2_/N-MWCNT composite material for supercapacitor applications. The peaks marked in green at 28.50°, 37.30°, 42.80°, 56.80°, 59.40°, and 72.50° corresponding to the (100), (101), (111), (113), (200), (220), (202), (400), (211), (220), (330), (400), and (420) planes of MnO_2_, respectively, matched with the data in PDF file no.44-0141. The 2*θ* peaks marked in red at 30.50°, 32.50°, 35.50°, 38.70°, 48.80°, 53.25°, 58.65°, 61.50°, 66.20°, 68.10°, 72.40°, and 75.20° corresponded to (110) (111) (202) (112), (020), (202), (113), (311), and (004) planes, respectively. The obtained results agreed with those of the CuO (JCPDS file no. 72-0629) monoclinic structure in the composite materials. In this XRD pattern, CuO@MnO_2_ was decorated on the N-MWCNT surface. Therefore, the MnO_2_/CuO in the composite materials was recognized as MnO_2_ with cubic and monoclinic CuO structure^20,21^.

The Raman spectra of the composites are shown in Fig. 2b. The Raman shifts at ∼ 1343, ∼ 1570, and 2450 cm^−1^ were ascribed to the three distinct peaks of the N-MWCNT composite. The D band was assigned to the lattice defect of the phonon mode of vibration from the N-MWCNT surface. The G band signifies the C–C (vibrational modes) and double-degenerate phonon modes of E_2*g*_ symmetry. The G band is associated with the vibrational modes of the sp^2^-bonded carbon atoms in the graphitic layer from the N-MWCNT, whereas the D band is associated with the breathing mode of sp^2^ bonding, which is present in disordered graphite. We observed that N doping enhanced the D-band intensity of the composite sample. The peak intensity ratio between the D and G bands (ID/IG) was 1.05 for the pristine N-MWCNT [ref], and 1.28 and 1.30, respectively, for the composite samples. This D enhancement indicates that N doping produces lattice defects in the graphitic layers^11,22,23^. The lower wave region of the metal oxides was formed with less intense Ag, Bg1, and Bg2 vibration modes, as discussed previously for metal oxide materials^24–27^.

### Morphological properties of the CuO@MnO_2_ composite

**Figure 3:**
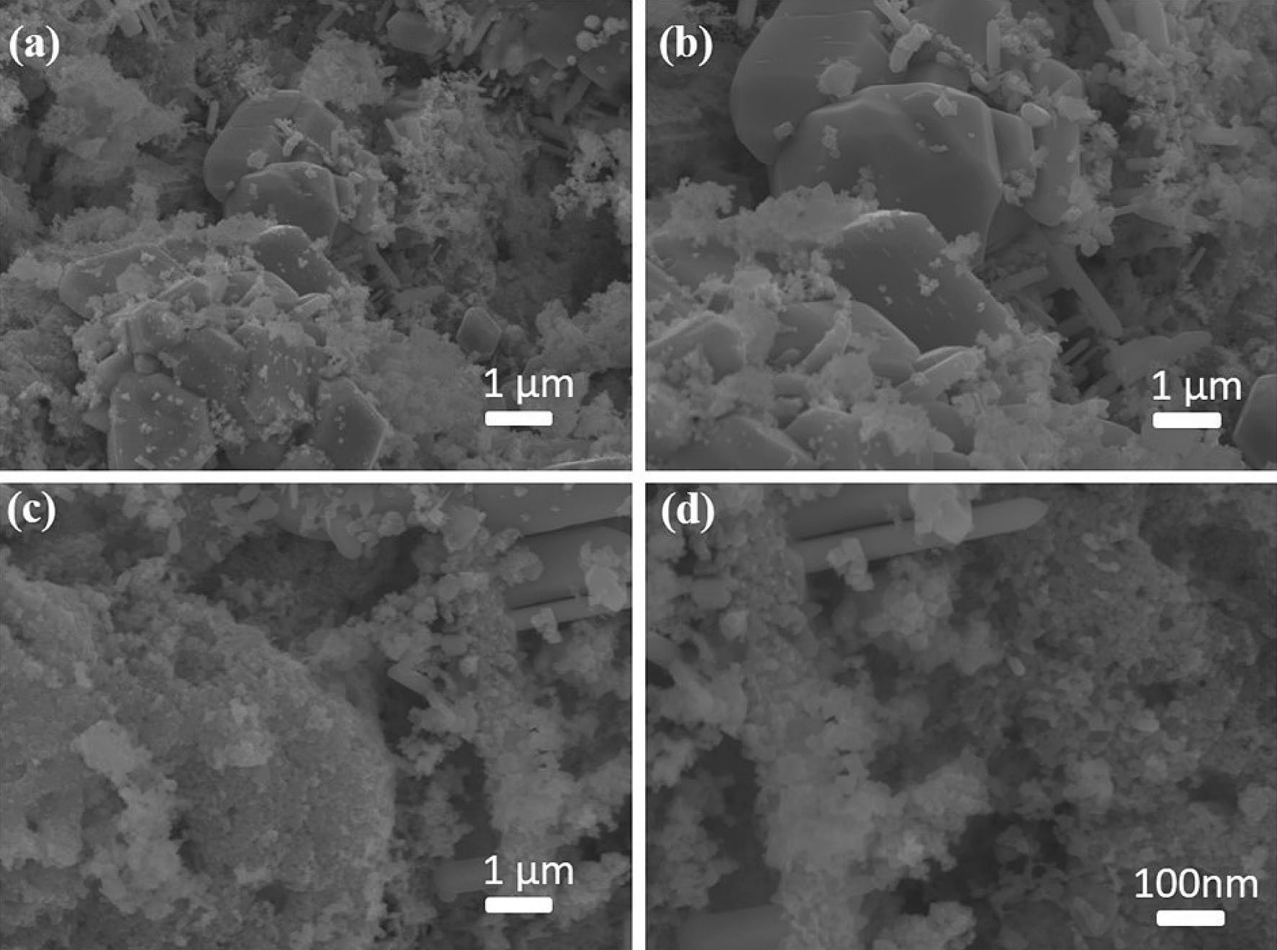
(**a–d**) SEM morphology of the CuO@MnO_2_ composite.

**Figure 4:**
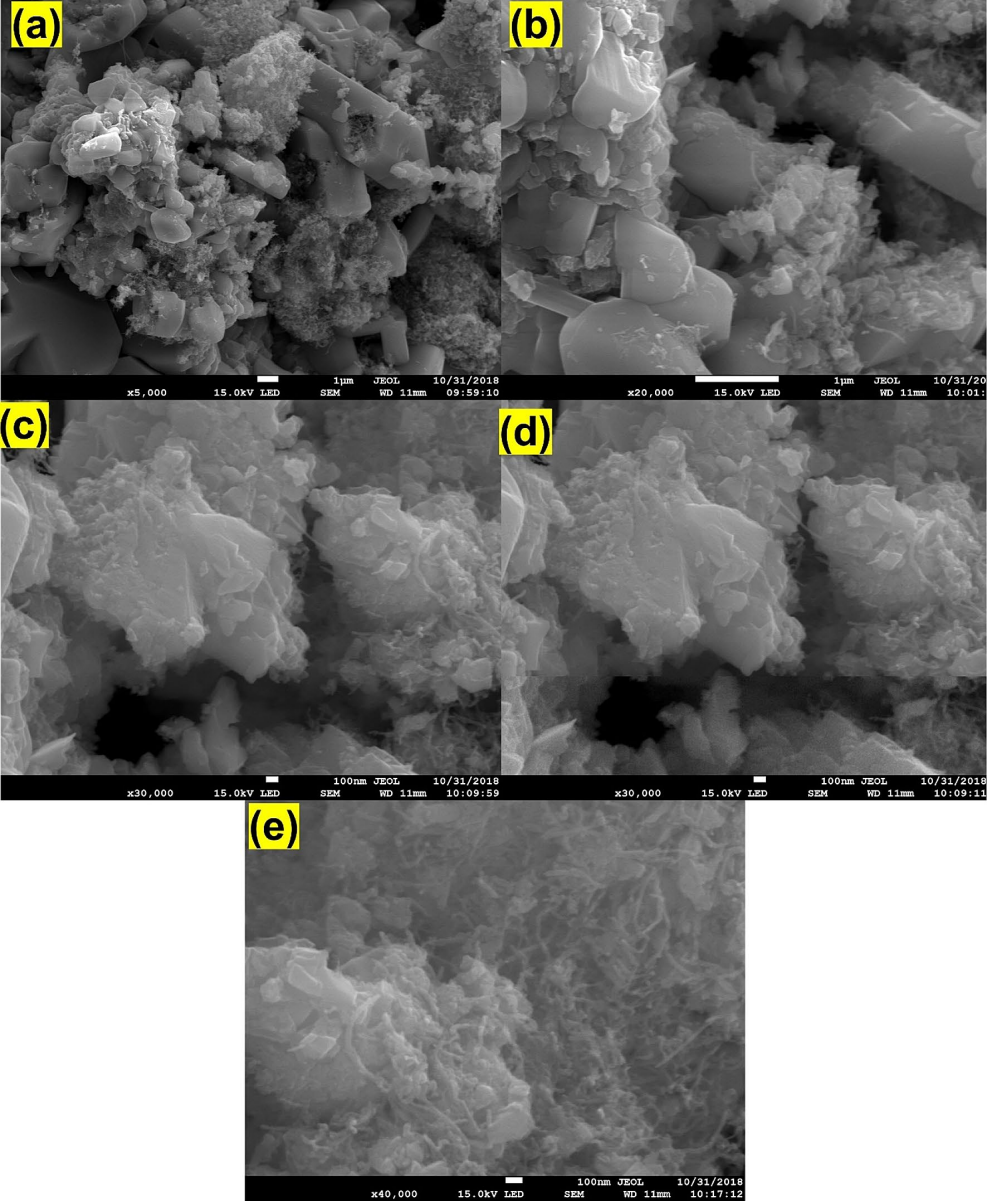
(**a–e**) SEM morphology of the CuO@MnO_2_/N-MWCNT composite.

The surface morphology of the composite was studied using SEM and SEM–EDS, and the results are shown in Figs. 3 and 4, respectively. The results revealed that the N-MWCNT tubes and CuO@MnO_2_ exhibited well-decorated nanotubes with an outer diameter of about ~ 20–30 nm and an inner thickness of ~ 10–20 nm (Fig. 4e). The SEM–EDS morphology of the composite is shown in (Supplementary Fig. S1). The results confirmed that C, O, N, Cu, and MnO_2_ were present in the synthesized composite materials. The morphological behavior of CuO- and MnO_2_-based materials has been previously reported in the literature^28–30^.

### Electrochemical properties of the composite electrodes

**Figure 5:**
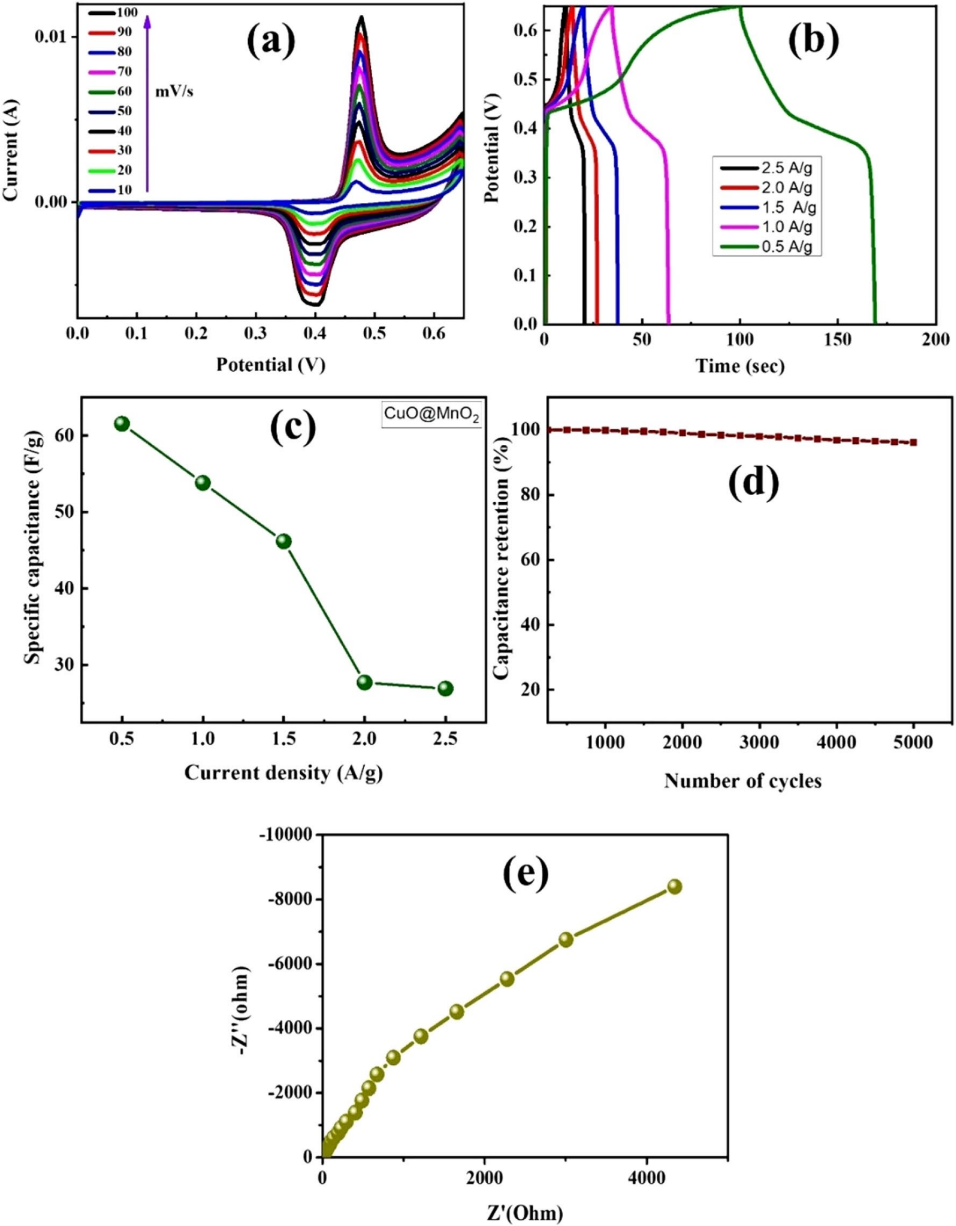
Results of electrochemical CV analysis. (**a**) CV; (**b**) GCD; (**c**) variation of specific capacitance with current density; (**d**) cyclic stability; and (**e**) EIS of the CuO@MnO_2_ composite electrode.

**Figure 6:**
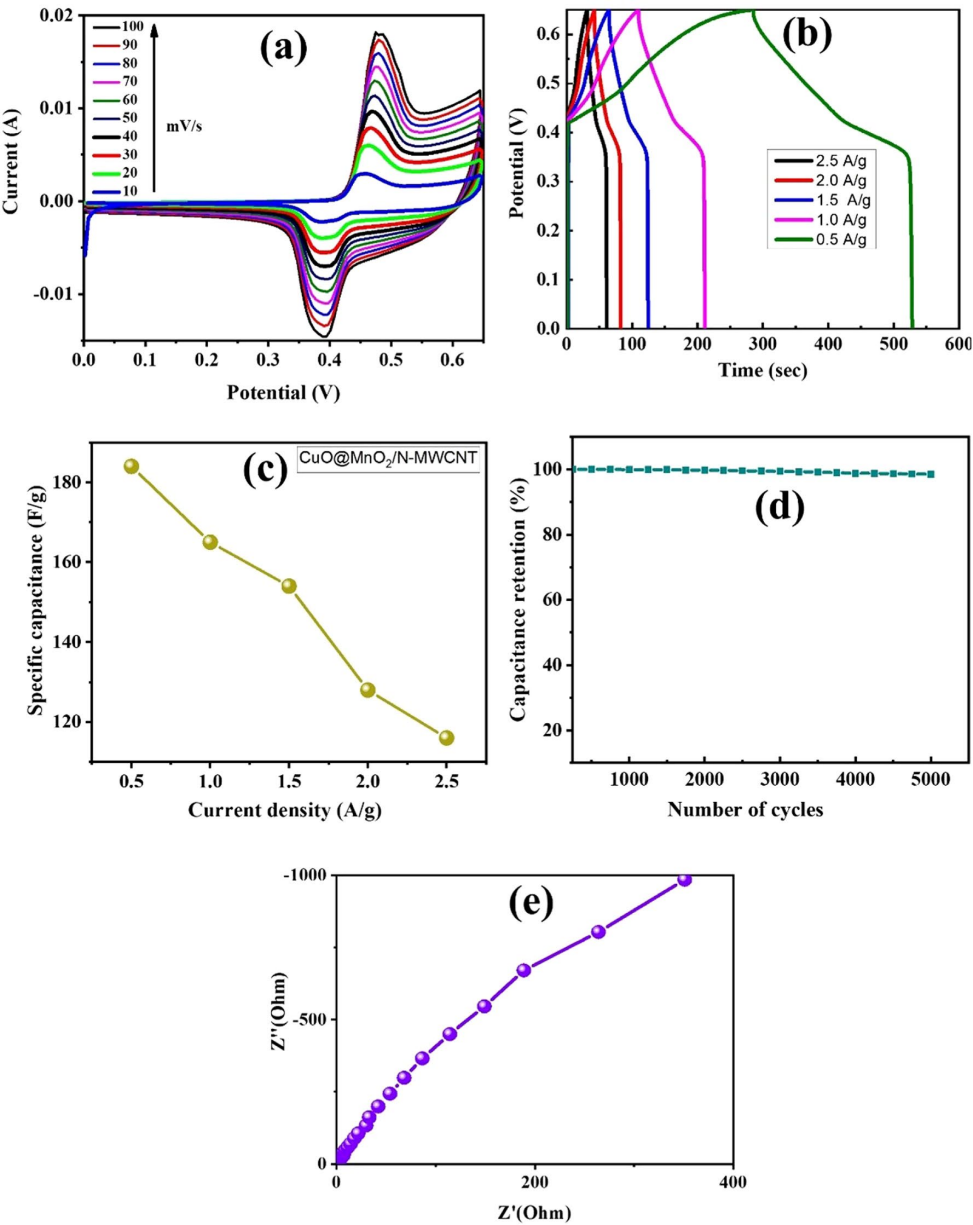
Results of electrochemical CV analysis. (**a**) CV; (**b**) GCD; (**c**) variation of specific capacitance with current density; (**d**) cyclic stability; and (**e**) EIS of the CuO@MnO_2_/N-MWCNT composite electrode.

The electrochemical properties of MnO_2_ and CuO oxides, and carbon-based materials have been investigated in several studies^31,32^, as these materials have potential electrode properties and cyclic stability, rendering them suitable for supercapacitor applications. In this study, the fabrication of CuO@MnO_2_ and CuO@MnO_2_/N-MWCNT composites was examined using a three-electrode configuration in the presence of a 5 M KOH electrolyte, and the results are shown in Figs. 5 and 6. The CV results for the composite are presented in Figs. 5 and 6a. The redox peaks were clearly observed for the CuO@MnO_2_ and CuO@MnO_2_/N-MWCNT composites. The excellent redox behavior of the composites was attributed to the reversible redox reaction between the active electrode materials and the electrolyte. The altered scan rates applied from the (10 to 100) mV/s results indicate that a similar trend of the electrochemical behaviors and rate capabilities occurred in the presence of the 5 M KOH electrolyte. The obtained results became more pronounced with scan rates and peak shifts toward positive and negative potentials owing to the polarization effect and electron transfer rates between the Cu and Mn oxides on the carbon surface^33–37^. The improved electrochemical behavior was strongly related to the chemical composition and morphology of the synthesized composite or 5 M KOH electrolyte for supercapacitor applications^38,39^.

GCD analysis was performed at current densities of 0.5, 1.0, 1.5, 2 and 2.5 A g^−1^ in the 0.0–0.6 V range, and the results are shown in Figs. 5b and 6b. The specific capacitance values were calculated using the equations reported in a previous study^40,41^. The composite electrodes exhibit triangular forms, which indicate reversible redox reactions in the electrochemical process. The outcome capacitance values of CuO@MnO_2_ were calculated as 61.54, 53.8, 46.15, 27.69 and 26.90 F g^−1^ at current densities of 0.5, 1.0, 1.5, 2, and 2.5 A g^−1^, respectively. The CuO@MnO_2_/N-MWCNT composite yields values of 184, 165,154, 125, and 116 F g^−1^ at the same current density. The variation of specific capacitance with current density is shown in Figs. 5c and 6c. The increase in specific capacitance was almost threefold owing to the interaction of the metal oxide nanoparticles with the nitrogen-doped carbon nanotubes. The cyclic stabilities of the composite electrodes prepared from the composites are shown in Figs. 5d and 6d. This result reveals that the GCD test was performed at a current density of 0.5 A g^−1^ for 5000 cycles. The specific capacitance of the composite electrode was 98.5% of its initial value, indicating the excellent stability retention and the performance of the composite electrode materials in terms of enhanced electrochemical properties with a 5 M KOH electrolyte. The unique morphologies of the synthesized composite materials may prevent aggregation and volume expansion during long-term cycling, which is useful for achieving structural stability of the electrodes^42,43^.

The results revealed that the synthesized composites exhibited superior specific capacitance values compared with those fabricated in previous studies (Table 1). The improvement in the specific capacitance of the CuO@MnO_2_ and CuO@MnO_2_/N-MWCNT composite electrodes resulted from the surface properties and improved morphological properties of the electrodes. The specific capacitances of the CuO@MnO_2_ and CuO@MnO_2_/N-MWCNT composite electrode materials were better than those of other electrodes with previously reported materials, as shown in Table 1^42,43^. The improvement in the electrochemical properties depends on the Faradaic redox reactions of the Cu (I)/Cu(II) and Mn (Mn^2+^) transitions and increases the capacitances, facilitating supercapacitor applications^43,44^. Furthermore, the electrochemical behaviors of the synthesized CuO@MnO_2_ andCuO@MnO_2_/N-MWCNT composite electrodes were studied via impedance spectroscopy (EIS) analysis, and the fabricated electrodes of composite (EIS) results are presented in Figs. 5e and 6e. The composite showed excellent electrochemical properties in the lower frequency region in the presence of the electrolyte, indicating that the electrochemical capacitive behavior of the composite electrode materials determines the parameters that affect the electrochemical performances^45–47^. Furthermore, the Cu2+/Cu+ and Mn2+ to Mn+ redox reactions improve the electrochemical behavior of the electrode materials in the lower wave frequency region, making these composites potentially suitable for supercapacitor applications^48–53^.

**Table 1:** Comparison of the electrochemical properties of the synthesized CuO@MnO_2_ and CuO@MnO_2_/N-MWCNT composite with those of composite materials reported in the literature.

Synthesized composites	Fabrication methods	Capacitance (F g^−1^)	Cyclic stability	Ref.
Cu_2_O/MOF carbon composite	Simple one step polyol method	151 F g^−1^ at 1	10% loss after 2500 cycles	^49^
CuO/NIO/RGO composite	Sonication assisted solvothermal process	395 F g^−1^ at 0.5 A g^−1^	11% loss after 5000 cycles	^50^
MWCNT/NiO/PPY composite	Thermal reduction process	239.5 F g^−1^ at 0.5 A g^−1^	2.68% loss after 1000 cycles	^51^
MnO_2_/CuO core shell materials	One step hydrothermal process	167.2 F g^−1^ at 0.3 A g^−1^	11.4% loss after 5000 cycles	^52^
Leaf like CuO-Cu_2_O electrode	One step anodization method	1.954 F cm^−2^ at 2 A.g^−1^	19.5% loss after 2000 cycles	^53^
Flower like CuO	Chemical precipitation method	133.6 F g^−1^ at 2 A g^−1^	5.2% loss after 200 cycles	^54^
MnO_2_/CNT/CP composite	Chemical vapor deposition	200 F g^−1^ at 1 m A/cm^2^	1% loss after 1000 cycles	^55^
CuO@MnO_2_/N-MWCNT composite	Sonication supported hydrothermal synthesis	184 F g^−1^ at 0.5A g^−1^	1.5% loss after 5000 cycles	Current study

## Conclusion

CuO@MnO_2_ and CuO@MnO_2_/N-MWCNT composites synthesized via a sonication-assisted process and hydrothermal reaction process exhibited excellent morphological properties and improved electrochemical behavior. The moreover, the CuO@MnO_2_/N-MWCNT composite electrodes exhibited excellent electrochemical properties with a high specific capacitance of 184 F g^−1^ at a current density of 0.5 A g^−1^ in the presence of 5 M KOH electrolytes. In particular, a capacity retention of 98.5% at 0.5 A g^−1^ was maintained over 5000 continuous GCD cycles. This enhancement in the electrochemical properties of the material was attributed to the surface properties and improved morphological behavior of the material. The synthesized composite electrodes exhibited excellent electrochemical, morphological, and cyclic stabilities, rendering them suitable for supercapacitor applications.

## Supplementary Information

Supplementary Figure S1.

## Data Availability

The datasets used and/or analyzed during the current study are available from the corresponding author on reasonable request.

## References

[1] Lou XW, Archer LA, Yang Z (2008). Hollow micro-/nanostructures: Synthesis and applications. Adv. Mater..

[2] Wang G, Zhang L, Zhang J (2012). A review of electrode materials for electrochemical super-capacitors. Chem. Soc. Rev..

[3] Ouyang L, Hsiao C-H, Chen Y-C, Lee CY, Tai NH (2021). Fabrication of Ni-Mn LDH/Co3O4 on carbon paper for the application in supercapacitors. Surf. Interface.

[4] Fan Z (2011). Asymmetric supercapacitors based on graphene/MnO_2_ and activated carbon nanofiber electrodes with high power and energy density. Adv. Funct. Mater..

[5] Ramesh S (2021). Core shell nanostructured of Co_3_O_4_@RuO_2_ assembled on nitrogen-doped graphene sheets electrode for an efficient supercapacitor application. J. Alloy. Compd..

[6] Racik M (2020). Fabrication of manganese oxide decorated copper oxide (MnO2/CuO) nanocomposite electrodes for energy storage supercapacitor devices. Physica E.

[7] Lei Z, Zhang J, Zhao XS (2012). Ultrathin MnO_2_ nanofibers grown on graphitic carbon spheres as high-performance asymmetric supercapacitor electrodes. J. Mater. Chem. A.

[8] Ramesh S (2019). Nanostructured CuO/Co_2_O_4_@ nitrogen doped MWCNT hybrid composite electrode for high-performance supercapacitors. Compos. B Eng..

[9] Bathula C, Rabani I, Ramesh S (2021). Highly efficient solid-state synthesis of Co_3_O_4_ on multiwalled carbon nanotubes for supercapacitors. J. Alloy. Compd..

[10] Li Q (2012). Design and synthesis of MnO_2_/Mn/MnO_2_ sandwich-structured nanotube arrays with high super capacitive performance for electrochemical energy storage. Nano Lett..

[11] Zhang H, Zhang M (2008). Synthesis of CuO nanocrystalline and their application as electrode materials for capacitors. Mater. Chem. Phys..

[12] Khan MA, Wahab Y, Muhammad R, Tahir M, Sakrani S (2018). Catalyst-free fabrication of novel ZnO/CuO Core-Shell nanowires heterojunction: Controlled growth, structural and optoelectronic properties. Appl. Surf. Sci..

[13] Ramesh S (2020). Fabrication of nanostructured SnO_2_@Co_3_O_4_/nitrogen doped graphene oxide composite for symmetric and asymmetric storage devices. J. Mater. Res. Technol..

[14] Ramesh S, Karuppasamy K, Kim HS, Kim HS, Kim J-H (2018). Hierarchical flowerlike 3D nanostructure of Co_3_O_4_@MnO_2_/N-doped graphene oxide (NGO) hybrid composite for a high-performance supercapacitor. Sci. Rep..

[15] Zhang YX, Li F, Huang M (2013). One-step hydrothermal synthesis of hierarchical MnO2-coated CuO flower-like nanostructures with enhanced electrochemical properties for supercapacitor. Mater. Lett..

[16] Feng Q, Kanoh H, Ooi K (1999). Manganese oxide porous crystals. J. Mater. Chem..

[17] Zhou Y, Cheng X, Tynan B, Sha Z, Huang F, Islam MS (2021). High-performance hierarchical MnO_2_/CNT electrode for multifunctional supercapacitors. Carbon.

[18] Toupin M, Brousse T, Bélanger D (2004). Charge storage mechanism of MnO_2_ electrode used in aqueous electrochemical capacitor. Chem. Mater..

[19] Gueon D, Moon JH (2015). Nitrogen-doped carbon nanotube spherical particles for supercapacitor applications: Emulsion-assisted compact packing and capacitance enhancement. ACS Appl. Mater. Interfaces.

[20] Huang M, Li F, Dong F, Zhang YX, Zhang LL (2015). MnO_2_-based nanostructures for high-performance supercapacitors. J. Mater. Chem. A.

[21] Zhang Y, Guo WW, Zhang ZTX, Fan YX (2018). Engineering hierarchical Di-atom@CuO@MnO2 hybrid for high performance supercapacitor. Appl. Surf. Sci..

[22] Guo XL, Li G, Kuang M, Zhang YL (2015). Tailoring kirkendall effect of the KCu_7_S_4_ microwires towards CuO@MnO_2_ core-shell nanostructures for supercapacitors. Electrochim. Acta.

[23] Wang JG, Huang YY, Kang ZH (2012). Synthesis and electrochemical performance of MnO_2_/CNTs-embedded carbon nanofibers nanocomposites for supercapacitors. Electrochim. Acta.

[24] Chavhan J, Rathod R, Tandon V, Umare S, Patel A (2022). Structural and physico-chemical properties of electroactive polyamide/multi-walled carbon nanotubes nanocomposites. Surf. Interfaces.

[25] Deng MJ (2014). Facile electrochemical synthesis of 3D nano-architecture CuO electrodes for high-performance supercapacitors. J. Mater. Chem. A.

[26] Li Y (2012). Nanostructured CuO directly grown on copper foam and their supercapacitance performance. Electrochim. Acta.

[27] Wang G, Huang J, Chen S, Gao Y, Cao D (2011). Preparation and supercapacitance of CuO nanosheet arrays grown on nickel foam. J. Power Sources.

[28] Subramanian V, Zhu H, Wei B (2006). Nanostructured MnO2: Hydrothermal synthesis and electrochemical properties as a supercapacitor electrode material. Power Source.

[29] Mishra AK, Das NAK, Pradhan AK (2018). Microwave-assisted solvothermal synthesis of cupric oxide nanostructures for high-performance supercapacitor. J. Phys. Chem. C.

[30] Deng L (2013). Preparation and capacitance of graphene/multiwall carbon nanotubes/MnO_2_ hybrid material for high-performance asymmetrical electrochemical capacitor. Electrochim. Acta.

[31] Yu L, Zhang G, Yuan C, Lou X (2013). Hierarchical NiCo_2_O_4_@MnO_2_ core-shell heterostructure nanowire arrays on Ni foam as high-performance supercapacitor electrodes. Chem. Commun..

[32] Wei W, Cui X, Chen W, Ivey DG (2011). Manganese oxide-based materials as electrochemical super-capacitor electrodes. Chem. Soc. Rev..

[33] Liu J (2011). Co_3_O_4_ nanowire@MnO_2_ ultrathin nanosheet core/shell arrays: A new class of high-performance pseudocapacitive materials. Adv. Mater..

[34] Guo CX, Wang M, Chen T, Li LXW, Li CM (2011). A hierarchically nanostructured composite of MnO2/conjugated polymer/graphene for high-performance lithium-ion batteries. Adv. Energy Mater..

[35] Zhang Y (2012). Crystallization design of MnO_2_ towards better supercapacitance. Cryst. Eng. Commun..

[36] Kim H, Popov BN (2003). Synthesis and characterization of MnO_2_-based mixed oxides as supercapacitors. J. Electrochem. Soc..

[37] Gholivand MB, Heydari H, Abdolmaleki A, Hosseini H (2015). Nanostructured CuO/PANI composite as supercapacitor electrode material. Mater. Sci. Semicond. Process.

[38] Zhao Y, Jiang P, Xie SS (2013). ZnO-template-mediated synthesis of three-dimensional coral-like MnO2 nanostructure for supercapacitors. J. Power Sources.

[39] Kumar R, Manoj D, Santhanalakshmi DJ (2013). Optimization of site-specific adsorption of oleylamine capped CuO nanoparticles on MWCNTs for electrochemical determination of guanosine. Sens. Actuator B Chem..

[40] Zhi M, Manivannan A, Meng F, Wu N (2012). Highly conductive electrospun carbon nanofiber/MnO_2_ coaxial nano-cables for high energy and power density supercapacitors. J. Power Sources.

[41] Ramesh S (2021). Sheet-like morphology CuCo_2_O_4_ bimetallic nanoparticles adorned on graphene oxide composites for symmetrical energy storage applications. J. Alloy. Compd..

[42] Pendashteh A, Mousavi MF, Rahmanifar MS (2013). Fabrication anchored copper oxide nanoparticles on graphene oxide nanosheets via an electrostatic co-precipitation and its application as supercapacitor. Electrochim. Acta.

[43] Palem RSRR (2021). Enhanced super capacitive behavior by CuO@MnO_2_/carboxymethyl cellulose composites. Ceram. Int..

[44] Zhang YX (2013). Facile synthesis of mesoporous CuO nanoribbons for electrochemical capacitors applications. Int. J. Electrochem. Sci..

[45] Haldorai Y, Voit W, Shim J-J (2014). Nano ZnO @ reduced graphene oxide composite for high performance supercapacitor: Green synthesis in supercritical fluid. Electrochim. Acta.

[46] Ramesh KS (2021). Hierarchical Co_3_O_4_ decorated nitrogen-doped graphene oxide nanosheets for energy storage and gas sensing applications. J. Ind. Eng. Chem..

[47] Purushothaman KK, Saravanakumar B, Babu IM, Sethuraman B, Muralidharan G (2014). Nanostructured CuO/reduced graphene oxide composite for hybrid supercapacitors. RSC Adv..

[48] Ramesh S (2021). Hexagonal nanostructured cobalt oxide @nitrogen doped multiwalled carbon nanotubes/polypyyrole composite for supercapacitor and electrochemical glucose sensor. Colloids Surf. B Bio Interfaces.

[49] Yadav HM (2019). Nanorods to hexagonal nanosheets of CuO-doped manganese oxide nanostructures for higher electrochemical supercapacitor performance. Colloids Surf. B Biointerfaces.

[50] Karuppasamy K (2020). Highly porous, hierarchical microglobules of Co_3_O_4_ embedded N-doped carbon matrix for high performance asymmetric supercapacitors. Appl. Surf. Sci..

[51] Reddy ALM, Ramaprabhu S (2007). Nanocrystalline metal oxides dispersed multiwalled carbon nanotubes as supercapacitor electrodes. J. Phys. Chem. C.

[52] Kathalingam A (2020). Nanosheet-like ZnCo_2_O_4_@nitrogen doped graphene oxide/polyaniline composite for supercapacitor application: Effect of polyaniline incorporation. J. Alloy. Compds..

[53] Liu C, Li F, Cheng MLP (2010). Advanced materials for energy storage. Adv. Mater..

[54] Abdullah A, Parveen N, Ahmad F, Alam MW, Ansari SA (2019). Self-assembled cube-like copper oxide derived from a metal-organic framework as a high-performance electrochemical supercapacitive electrode material. Sci. Rep..

[55] Kakani V (2020). Facile synthesis of CuO/NiO/nitrogen doped rGO by ultrasonication for high performance supercapacitors. J. Alloy. Compd..

