# Supplementary material for: Hydrothermal synthesis of CuO@MnO2 on nitrogen-doped multiwalled carbon nanotube composite electrodes for supercapacitor applications
Source: Sci Rep. 2022 Sep 20;12:12951. doi: 10.1038/s41598-022-16863-3 (PMC9489798; doi:10.1038/s41598-022-16863-3)
Supplement: Supplementary file 1 — Supplementary Figure S1. [file 41598_2022_16863_MOESM1_ESM.docx]

**SUPPLEMENTARY INFORMATION**

**Hydrothermal synthesis of CuO@MnO2 on nitrogen-doped multiwalled carbon nanotube composite electrodes for supercapacitor applications**

Vijay Kakani1, Sivalingam Ramesh2, H.M. Yadav3, Chinna Bathula4, Praveen Kumar Basivi5, Ramasubba Reddy Palem6, Heung Soo Kim2, Visweswara Rao Pasupuletti7*,*8, Handol Lee9*,∗* & Hakil Kim10*,∗*


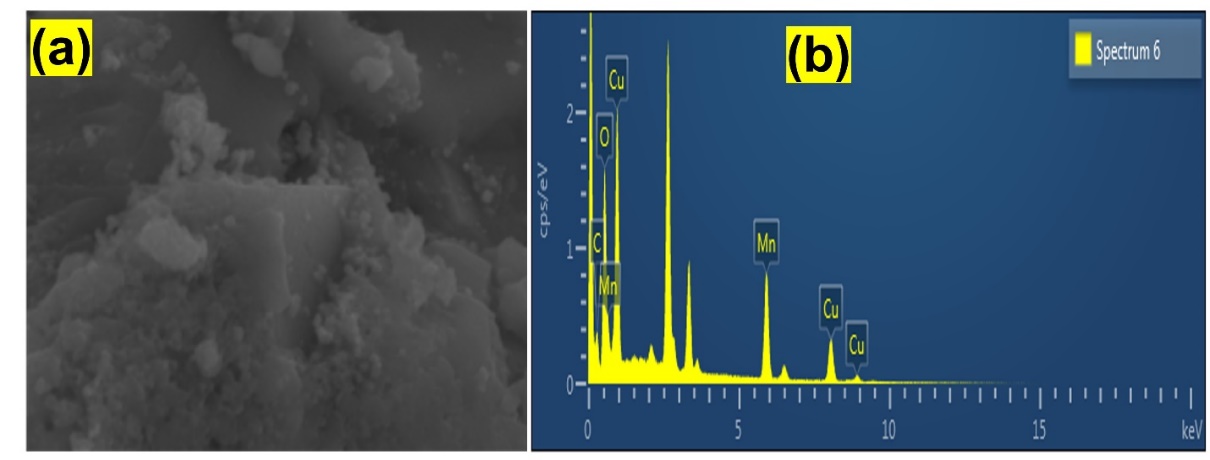


**Figure.S1.** (**a-b**) SEM-EDS morphology and composition of CuO@MnO_2_/N-MWCNT composite.
